# Scalable analysis of large multi-ancestry biobanks by leveraging sparse ancestry-adjusted sample-relatedness

**DOI:** 10.21203/rs.3.rs-5343361/v1

**Published:** 2024-11-12

**Authors:** Xihong Lin, Rounak Dey, Xihao Li, Zilin Li

**Affiliations:** Harvard T.H. Chan School of Public Health; Insitro, Inc; University of North Carolina at Chapel Hill; Northeast Normal University

## Abstract

Linear mixed-effects models (LMMs) and ridge regression are commonly applied in genetic association studies to control for population structure and sample-relatedness. To control for sample-relatedness, the existing methods use empirical genetic relatedness matrices (GRM) either explicitly or conceptually. This works well with mostly homogeneous populations, however, in multi-ancestry heterogeneous populations, GRMs are confounded with population structure which leads to inflated type I error rates, massively increased computation, and reduced power. Here, we propose FastSparseGRM, a scalable pipeline for multi-ancestry Genome-Wide Association studies (GWAS) and Whole Genome Sequencing (WGS) studies. It utilizes a block-diagonal sparse ancestry-adjusted (BDSA) GRM to model sample-relatedness, and ancestry PCs as fixed effects to control for population structure. It is ~ 2540/4100/54 times faster than BOLT-LMM/fast-GWA/REGENIE for fitting the null LMM on 50,000 heterogeneous subjects. Through numerical simulations and both single-variant GWAS and rare variant WGS analyses of five biomarkers (Triglycerides, HDL, LDL, BMI, Total Bilirubin) on the entire UK Biobank data, we demonstrate that our approach scales to nearly half-a-million subjects and provides accurate p-value calibration and improved power compared to the existing methods.

## Introduction

Modern large cohort studies and biobanks^1,2^ provide a landscape of opportunities to discover genetic components of complex human traits through large-scale genome-wide and phenome-wide association studies (GWAS or PheWAS) for common variant effects and Whole Genome Sequencing (WGS) Studies^3^ for rare and common variant effects. Even though most of the published GWASs in the past decade have disproportionately focused on participants of Caucasian ancestry^4^, more recently there has been a substantial effort to recruit participants from diverse ancestral backgrounds in multi-ancestry studies and biobanks, such as the UK Biobank^1^, Trans-Omics for Precision Medicine (TOPMed)^2^, Million Veteran Program (MVP)^5^, and All of US^6,7^. For instance, TOPMed has 45% European, 26% Black, 21% Hispanic, 7% Asian ancestry subjects, and All of Us has 52% European, 18% Black, 17% Hispanic, 3% Asian ancestry subjects. About 1/3rd of the UK Biobank and TOPMed samples are related. In presence of such strong ancestral heterogeneity and sample-relatedness, the existing genetic association testing methods based on the traditional genetic relatedness Matrices (GRMs) such as BOLT-LMM^8^ and fastGWA^9^, cannot appropriately disentangle the ancestry and relatedness, which can potentially make them computationally challenging to analyze large GWAS and WGS biobanks, and also affect the p-value calibration (controlled type I error) and power, especially for multi-ancestry genetic studies. In this paper, we propose a scalable and accurate generalized linear mixed model (GLMM) based genetic association testing pipeline to address emerging challenges of analysing large-scale multi-ancestry biobank data.

Over the past decade, application of linear mixed models (LMMs) has become the mainstream approach to account for population structures and known or unknown relatedness among the individuals in GWASs^8,10–12^ and WGS studies^13,14^. To achieve valid statistical inference, LMMs commonly use ancestry principal components^15^ (PCs) and genetic relatedness matrices^16^ (GRMs) especially in studies where subjects come from diverse ancestries or where sample relatedness is mostly unknown. Calculating the PCs and GRMs to model the genome-wide population structure and relatedness comes at a high computational cost. For example, a GRM for N=500,000 subjects would require at least 931 GB of storage $\left(O\left(N^{2}\right)\right)$ in upper-triangular dense 64-bit format and $\left(O\left(N^{2}M\right)\right)$ computation, where M is the number of variants used to compute the GRM. These large GRMs will also burden their usage in the association analysis which include matrix multiplications and matrix inversions $(O\left(N^{2}\right)$ and $O\left(N^{3}\right)$ computation, respectively), potentially making them infeasible. Table 1 shows a schematic structure of different steps, computation complexities, and memory and storage requirements of different state-of-the-art methods in running the entire GWAS pipeline.

In order to address this memory and storage problem, fastGWA uses a sparse version of the GRM in mixed models^9,17^, which offers a good degree of sparsity for homogenous samples. Using a sparse GRM with $S^{2}$ non-zero elements in the fastGWA^9^ method requires $O\left(S^{2}+N\right)$ computation and $O\left(S^{2}+N\right)$ memory to fit the LMM. However, in multi-ancestry studies, the sparse GRM from fastGWA can be confounded with population structure^18^, where each block corresponds to an ancestry group and can be considerably dense, especially when sample sizes in different ancestries are not small, such as TOPMed and All of Us (Fig. 2). This lack of sparsity increases the computation time, potentially making it infeasible to be applied on large multi-ancestry biobanks, as well as affecting type I error rate control and power (Figs. 5 and 6).

**Figure 1:**
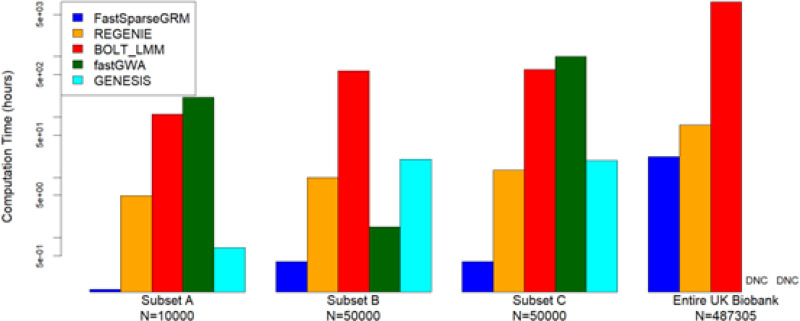
Computation time to fit the LMM/RR (including computing any required PCs and GRM) for 1500 phenotypes using analysis pipelines of FastSparseGRM, REGENIE, BOLT-LMM, fastGWA, and GENESIS: All computations were performed on 30 CPUs. For each method, 100 simulated phenotypes were analyzed, and the resource requirements were projected for 1500 phenotypes. REGENIE was applied on 100 phenotypes together in one run, whereas the other methods were applied on each phenotype sequentially. Four different subsampled datasets from the UK Biobank data were used (see Results). For the entire UK Biobank dataset, fastGWA and GENESIS did not complete (DNC) the analysis within the 5-day, 1.5TB constraint. The y-axis is in logarithmic scale.

GENESIS^17^, on the other hand, calculates a block-diagonal sparse GRM by thresholding the dense GRM produced by the PCRelate^18^ method, which results in a reduced computation time requirement of $O\left(L^{2}+N\right)$ for fitting the LMM, where L is the order of the largest related sample-block. Even though the usage of this sparse GRM in LMMs is attractive for the computational efficiency, the GENESIS computation of the sparse GRM itself entails extremely high computational burden. Since it requires estimating a dense GRM first, the extremely high computational $\left(O\left(N^{2}M\right)\right)$ and memory $\left(O\left(N^{2}\right)\right)$ costs create a bottleneck in the analysis pipeline, potentially making it infeasible for large biobanks and WGS analyses (Fig. 1).

BOLT-LMM^8^ and REGENIE^19^ circumvent the GRM storage problem by performing the necessary computation of genetic relatedness on-the-fly during the model fitting procedure. BOLT-LMM calculates the GRM elements iteratively using the raw genotype data stored in 2-bit format and performs matrix inversions using the pre-conditioned conjugate gradient^20^ (PCG) method, reducing the memory requirement to O(NM), and achieving an effective computation time of $O\left(N^{1.5}M\right)$ to fit the LMM, which is not linear in *N* and computationally remains costly for large-scale multi-ancestry biobanks. On the other hand, REGENIE fits a ridge regression (RR) with genome-wide SNPs as covariates, achieving a computation time and memory requirement of $O\left(NMB+MB^{2}+N{\left(\frac{M}{B}\right)}^{2}+{\left(\frac{M}{B}\right)}^{3}\right)$ and O(NM) respectively in their RR fitting step, where B is the number of SNPs in each block. Mathematically, one can show that RR is equivalent to LMM where the ridge penalty can be interpreted as the inverse of the variance component parameter.

Even though BOLT-LMM and REGENIE are computationally scalable to analyze large biobanks, the LMM/RR models in these methods do not appropriately account for the population structure and sample-relatedness in multi-ancestry studies, such as TOPMed and All of Us, that have substantial population structure especially in the presence of admixture samples. Specifically, BOLT-LMM does not use PCs as fixed effects. In absence of PCs as fixed effects, the GRM is inadequate to control for strong population structure, which can potentially lead to an inflation of false positive rates^21^ (Fig. 5). On the other hand, REGENIE uses PCs as unregularized covariates (RR-equivalent of fixed effects) and genome-wide SNPs as regularized covariates (RR-equivalent of random effects). Since genome-wide SNPs include all the information present in the PCs, this results in a *double-fitting* of the population structure, both as fixed effects (unregularized) and also as random effects (regularized), which can reduce the power of the test (Fig. 6). The same pitfall applies if one extends BOLT-LMM by including PCs as fixed effects.

Additionally, all of these methods, except BOLT-LMM, suggest using PCs as fixed effect covariates to account for the ancestral heterogeneity. These methods also treat the calculation of PCs to be a pre-processing step separate from the main GWAS pipeline, i.e., users are required to input pre-calculated PCs as part of covariates, which is inconvenient, except for GENESIS which calculates the PCs using the PCAiR^22^ method within the pipeline. The calculation of PCs incurs an additional $O\left(\min\left(N^{2}M,NM^{2}\right)\right)$ (for exact PCs) or O(NM) (for randomized PCs^23–25^) computation to the analysis pipeline. To the best of our knowledge, no existing PCA implementation to estimate PCs from genetic data can take advantage of modern computing clusters and utilize parallel computing efficiently to reduce this user time burden. All methods discussed above for fitting LMM uses parallel computing in other steps of the analysis, making the single-CPU estimation of PCs a crucial bottleneck.

To address the aforementioned computational and statistical challenges, in this paper, we propose FastSparseGRM, an analysis pipeline that 1) efficiently estimates a block-diagonal sparse ancestry-adjusted (BDSA) GRM by disentangling the population structure from sample-relatedness, with the estimated blocks representing inferred families; 2) estimates ancestry PCs within the pipeline, removing any additional requirement to estimate them separately; 3) uses the ancestry PCs as fixed effect covariates to account for the population structure, and the sparse BDSA-GRM to account for the sample relatedness in an LMM framework to provide for scalable analysis of large-scale multi-ancestry GWAS/WGS studies and biobanks.

Specifically, our work makes the following contributions. Firstly, we provide an extremely efficient algorithm to estimate the BDSA-GRM that only requires $O\left(NM+L^{2}M\right)$ computation and $O\left(NM+L^{2}\right)$ memory, where N is the sample-size, M is the number of variants used to compute the GRM, and L is the size of the largest related sample-block of the BDSA-GRM. For instance, with 50,000 UK Biobank subjects, our algorithm reduces the computation time of estimating the GRM by ~ 99% and ~ 53% compared to GENESIS and fastGWA, respectively (Table 2 and Supplementary Table 1). On the entire UK Biobank data of 487,305 subjects, FastSparseGRM reduces the computation time by ~ 76% compared to fastGWA, and GENESIS is unable to compute the BDSA-GRM within a 5-day, 1.5TB, 30-CPU resource limit. Keep in mind, fastGWA only computes an unadjusted sparse GRM, not a BDSA-GRM, and BOLT-LMM and REGENIE do not compute any GRM. FastSparseGRM achieves this improvement by only calculating the entries in the GRM corresponding to the related pedigree blocks as determined by the KING^26^ identity-by-descent (IBD) segment method, thus bypassing the need to compute the entire high-dimensional dense GRM, which is required by GENESIS.

Secondly, we implemented an efficient parallel computing algorithm for the PC estimation within the BDSA-GRM estimation process, providing a fast and parallelizable method of estimating genetic PCs instead of estimating them separately as a pre-processing step and inputting them together with other covariates, which is required in BOLT-LMM, fastGWA, and REGENIE. The computation of 20 PCs from the entire UK Biobank dataset of 487,305 subjects using 124,202 variants (variant-filtering details in Online Methods) required only 31 minutes of user time on 30 CPUs and 32.18GB overall memory usage using FastSparseGRM. GENESIS was unable to compute the PCs at the UK Biobank scale within a 5-day 500-GB, 30-CPU limit.

Thirdly, the LMM framework used in the FastSparseGRM pipeline provides accurate p-value calibration to control for the type I error rate, as well as results in the highest empirical power overall in multi-ancestry studies with strong population heterogeneity and sample-relatedness compared with the existing methods, whereas BOLT-LMM, fastGWA, and REGENIE can lead to a miscalibration of p-values (inflated type I error rates) or loss of power (Figs. 5 and 6). Additionally, on multi-ancestry datasets, the LMM null model fitting procedure in FastSparseGRM is ~ 700–2500 times faster than BOLT-LMM, ~ 2300–4200 times faster than fastGWA, and ~ 6.5–55 times faster than REGENIE (computation time varies by sample-size, see Fig. 1 and Supplementary Table 1), thereby improving the computation massively in PheWASs where multiple phenotypes need to be analysed. Computation of BDSA-GRM and PCs) is not feasible for GENESIS at UK Biobank scale, whereas FastSparseGRM is scalable enough to compute the both PCs and BDSA-GRM efficiently.

Finally, we have applied our method on the entire UK Biobank dataset of 487,305 subjects and computed the BDSA-GRM to account for relatedness, which was practically infeasible for the existing methods. Our single-variant analysis of five biomarkers (Triglycerides, High-density lipoprotein - HDL, Low-density lipoprotein – LDL, Body Mass Index – BMI, and Total Bilirubin) demonstrate FastSparseGRM’s applicability in large-scale GWASs. Further, the same BDSA-GRM fits readily into the STAAR^13^ method as implemented in STAARpipeline^27^, and can be applied for multi-ancestry rare variant association studies (RVAS) as well for analyzing whole-genome sequencing (WGS) data. We demonstrate this by performing gene-centric analysis of the lipid traits (LDL, HDL, and Triglycerides) with 200K WGS data.

## Results

### Overview of methods

The FastSparseGRM pipeline involves two main steps for analysis of large multi-ancestry genetic studies and biobanks to account for relatedness and population structure: A) Calculating the BDSA-GRM and ancestry PCs at scale, and B) Association testing using LMM where the BDSA-GRM and ancestry PCs are used to account for the sample-relatedness and population structure, respectively.

#### A) Calculation of the BDSA-GRM and ancestry PCs

FastSparseGRM efficiently calculates the BDSA-GRM and ancestry PCs at scale for large whole genome data in five steps as follows (schematic representation at Fig. 7): 1) Apply KING^26^ IBD segment method to identify the related samples and use them to form pedigree blocks; 2) calculate ancestry divergence^22^ metrics for the subjects which belong to at least one related pair; 3) extract a subset of unrelated samples, 4) apply principal component analysis (PCA) on the set of unrelated samples and project the rest of the samples on the resulting PC spaces to obtain the ancestry PCs, 5) compute the ancestry-adjusted allele frequencies for the markers by regressing out the ancestry PCs from the genotypes, and subsequently compute the elements in the BDSA-GRM corresponding to only the related samples of the pedigree blocks estimated by KING and setting the other elements to zero. The entire process is accomplished by applying KING for step 1, and then applying R wrapper functions provided in our implementation for steps 2–4. Steps 1 through 3 allow the estimation of genetic pedigrees and the selection of an ancestrally-diverse unrelated set of subjects for calculating the PCs and the BDSA-GRM.

FastSparseGRM achieves extremely fast computation of the BDSA-GRM mainly through two major advancements. Firstly, it initially estimates each pedigree block (step 1), and then only computes the elements in the BDSA-GRM corresponding to those blocks (step 5), instead of processing a high-dimensional dense GRM first as done by GENESIS and then thresholding the elements. Secondly, ancestry PCs are calculated using a fast parallelized randomized PCA algorithm in step 4, thus eliminating the need to calculate PCs separately as input variables for the association analysis required by several methods, such as FastGWA and REGENIE. Our implementation is highly parallelizable and can take advantage of multi-threading in modern high-performance computing clusters, thus substantially reducing the user time. Detailed descriptions of each of these steps are provided in the Online Methods section.

#### B) Association testing via LMM

To test for genotype-phenotype associations, FastSparseGRM applies the following LMM framework,

Y=Xβ+Gγ+b+є,

for N subjects, where $Y={\left(Y_{1},\ldots ,Y_{N}\right)}^{⊤}$ is the N×1 trait outcome vector, $X=\left(X_{1}^{⊤},...,X_{N}^{⊤}\right)$ is the N×k covariate matrix that includes the intercept and the ancestry PCs, $G={\left(G_{1},\ldots ,G_{N}\right)}^{⊤}$ is the N×1 genotype vector for the variant being tested, β and γ are corresponding coefficient parameters, b is a random effect vector to model the familial relatedness among the subjects, and є is the random environmental effect. We assume that $\text{є}={\left({\text{є}}_{1},\ldots ,{\text{є}}_{N}\right)}^{⊤}∼N\left(0,\sigma^{2}I\right)$, and $b={\left(b_{1},\ldots ,b_{N}\right)}^{⊤}∼N\left(0,\tau^{2}\Phi \right)$, where I denotes the N×N identity matrix, Φ denotes the BDSA-GRM, and $\sigma^{2}$ and $\tau^{2}$ are corresponding variance component parameters.

We perform the analysis in two steps: 1) fit the null model without the target variant, 2) perform a score test for the association between the genotypes of the target variant and the phenotype using the parameter estimates from the null model. To achieve fast computation of the score test genome-wide, we use the variance-ratio approximation commonly used in BOLT-LMM^8^ and fastGWA^9^ for GWAS analysis, where the variance of the score statistic is first calculated assuming the subjects are independent, and then adjusted by a variance-ratio^28^ factor that is calculated based on a small number (100) of variants.

This LMM modelling approach allows us to separate the effects of the distant-ancestral similarities and the near-ancestral similarities. The *distant-ancestral similarities* correspond to population structure, whose effects are modelled using ancestry PCs as fixed effects, while the *near-ancestral similarities* correspond to family relatedness, whose effects are modelled using random effects with the BDSA-GRM covariance matrix. We assume that the distant-ancestry (population structure) is of low-rank or finite-rank, i.e., there are a small finite (does not grow with N) number of ancestral populations, and every individual’s distant-ancestry is a combination of those ancestral populations. In the GWAS literature, this distant-ancestral effect is found to be well-captured by the ancestry PCs, and due to the finite-rank nature of this effect. The ancestry PCs are traditionally modelled as fixed effect covariates. On the other hand, we expect the family relatedness to be of high-rank or non-finite-rank, i.e., the number of families to grow with the sample-size N but the size of the largest family block remains small. Thus it is reasonable to model this effect as a random effect represented by the BDSA-GRM. Since ancestry PCs are adjusted for in the construction of the BDSA-GRM, our approach is able to disentangle the two effects properly, which BOLT-LMM, fastGWA, and REGENIE cannot.

GENESIS^17^ uses a BDSA-GRM to account for the sample relatedness and ancestry PCs to account for the population structure in the software. However, it is computationally not scalable for large biobanks, as it requires first estimating a dense GRM matrix and thresholding its elements. In contrast, FastSparseGRM provides an extremely fast algorithm to construct the BDSA-GRM by only calculating the non-zero elements of the BDSA-GRM corresponding to the related samples of the estimated pedigrees from KING and computing ancestry PCs using efficient parallel computing techniques.

### Datasets used in this paper

We used the entire UK Biobank data^1^ (N=487,305 subjects) and its different subsets to evaluate our FastSparseGRM pipeline and compare its performance with the existing methods, including BOLT-LMM, fastGWA, GENESIS and REGENIE. First, we compared the computation costs of constructing a BDSA-GRM between FastSparseGRM and GENESIS. Since computing a BDSA-GRM at the UK Biobank scale using GENESIS was infeasible, for a fair comparison, we used three subsets of the UK Biobank data; **Subset A**: 10,000 randomly selected subjects, among which 4,000 had self-reported White Irish ancestry, 3,000 had self-reported African and Caribbean ancestry, and 3,000 had self-reported South Asian ancestry, **Subset B**: 50,000 randomly selected subjects with white British ancestry; **Subset C**: 50,000 randomly selected subjects from the subjects that do not have white British ancestry. Here, the white British ancestry label was determined by the UK Biobank researchers, and it combines self-reported and genetically determined ancestries. Next, we compared the computation costs of running the entire pipeline (including calculation of PCs and GRMs as needed) using FastSparseGRM, BOLT-LMM, fastGWA, REGENIE, and GENESIS using Subsets A, B, C as well as the entire UK Biobank dataset. Finally, we used Subset A to compare the p-value calibration and empirical power among FastSparseGRM, BOLT-LMM, fastGWA, and REGENIE.

### Computation and memory costs

First, we compared the computation cost of constructing the BDSA-GRM between the FastSparseGRM and GENESIS methods. We performed this comparison based on Subsets A, B, and C. Both methods used 30 CPU cores. For subset A, overall, GENESIS required ~ 40 minutes of computation time and ~ 61 GB memory, and for each of the subsets B and C, GENESIS required ~ 19 hours of computation time and 390 GB memory. On the other hand, FastSparseGRM required only ~ 3 minutes of computation time and ~ 7 GB memory for subset A, and ~ 9 minutes of computation time and ~ 35 GB memory for subsets B and C each (Table 2). The computation time (memory) reduction for FastSparseGRM compared to GENESIS were ~ 92% (88%) for subset A, and ~ 99% (91%) for each of the subsets B and C. Detailed step-by-step benchmarking of these methods are presented in Supplementary Table 1.

We note that, since GENESIS uses the kinship estimates from KING for the initial inference of relatedness, which can be inaccurate, it requires at least two iterations to accurately estimate the GRM. The results presented here includes two iterations of running the GENESIS method. We compared the elements of the BDSA-GRMs computed by FastSparseGRM and GENESIS for the subsets A, B, and C. The non-zero elements of the BDSA-GRMs were nearly identical ($R^{2}$ and slope almost equal to unity in all of the subsets), and the Jaccard’s distance and dissimilarity proportions for the coordinates of the thresholded elements were nearly zero between the two methods (Supplementary Fig. 1). This result suggests that even though FastSparseGRM required only a small fraction of the computation time and memory required by GENESIS, it still provided almost identical BDSA-GRM to GENESIS.

We note that the memory requirement for GENESIS scales at $O\left(N^{2}\right)$ rate, whereas the memory requirement scales linearly at O(NM) rate for FastSparseGRM (Table 1). This makes the computation of a BDSA-GRM using the GENESIS approach practically infeasible for the entire UK Biobank dataset of 487,305 samples. Therefore, we only applied the FastSparseGRM method for the purpose of calculating the BDSA-GRM for the entire UK Biobank dataset. The overall computation time was ~ 11 hours on 30 CPUs with peak memory usage of 113 GB. GENESIS was not scalable enough to handle such a massive dataset and the computation did not finish within a 5-day limit. In terms of storage, the 487,305 × 487,305 BDSA-GRM only required 124 MB storage, whereas a dense GRM of the same dimensions would require ~ 885 GB disk space to store, which is 7308 times more. This makes FastSparseGRM to be the only method that can calculate a BDSA-GRM for the entire UK Biobank dataset using reasonable computing resources.

Next, we compared the computation costs of fitting the null model and performing the genome-wide association testing for 1500 simulated phenotypes using FastSparseGRM, REGENIE, BOLT-LMM, fastGWA, and GENESIS. We used the Subsets A, B, C, and the entire UK Biobank dataset for this study. Across all subsets, FastSparseGRM and GENESIS resulted in the shortest computation time for fitting the null model due to their use of BDSA-GRMs (see Fig. 1 and Supplementary Table 1). GENESIS was in fact ~ 5–6 times faster than FastSparseGRM at this step since it does not calculate the variance-ratio factors, which is an additional computation FastSparseGRM performs to facilitate efficient computing in the genome-wide scan step. However, both of these methods were extremely fast compared to BOLT-LMM, fastGWA and REGENIE in the null LMM model-fitting (or whole-genome RR) step. For instance, with Subset C, FastSparseGRM was > 2500 times faster than BOLT-LMM, > 4100 times faster than fastGWA, and ~ 54 times faster than REGENIE in this step.

**Figure 2:**
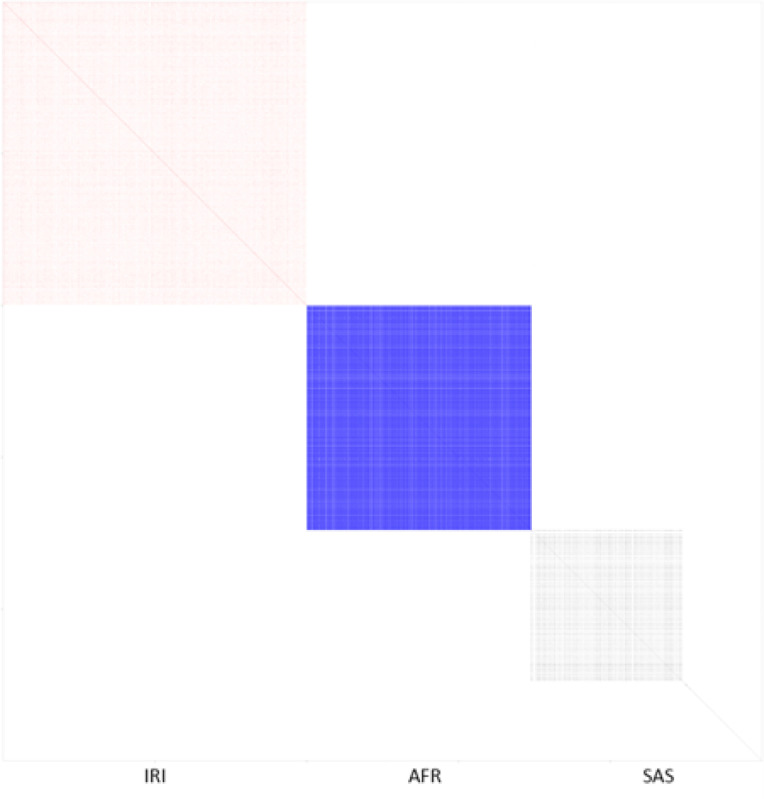
Sparse GRM based on the application of fastGWA on Subset A of the UK Biobank dataset with 10,000 subjects: Both the height and width of this image are of 10,000 pixels each, with each pixel representing whether a pair of subjects had their GRM coefficient thresholded to zero (white pixels) or not (colored pixels). Thresholding was performed at 0.05. The red (IRI: White Irish), blue (AFR: African), and grey (SAS: South Asian) pixels represent non-zero GRM elements for pairs of individuals within the same ancestry group.

The computation time for fastGWA was highly dependent on the ancestral diversity of the samples. For example, subsets B and C both were comprised of the same number of subjects (N = 50,000), but fastGWA required > 800 times more computation time for subset C compared to subset B. This can be attributed to the fact that the sparse GRM used by fastGWA was substantially less sparse (~ 400 times) in subset C as fastGWA does not adjust for the population structure in its sparse GRM, resulting in its non-zero elements representing the population structure instead of the familial relatedness (Fig. 2).

For the genome-wide scan step, all methods except for GENESIS required similar computation time. GENESIS required ~ 30 times more computation time because it does not use any variance-ratio type factor to improve the computation time. The overall computation times (including the time to compute the BDSA-GRM) were similar between FastSparseGRM and REGENIE, with FastSparseGRM being ~ 1.2–1.6 times faster than REGENIE. However, the computation of the BDSA-GRM is a one-time computation, and the resulting BDSA-GRM can be reused in any number of GWASs or downstream applications, e.g., PheWAS.

### GWAS analysis of the UK Biobank data

**Figure 3:**
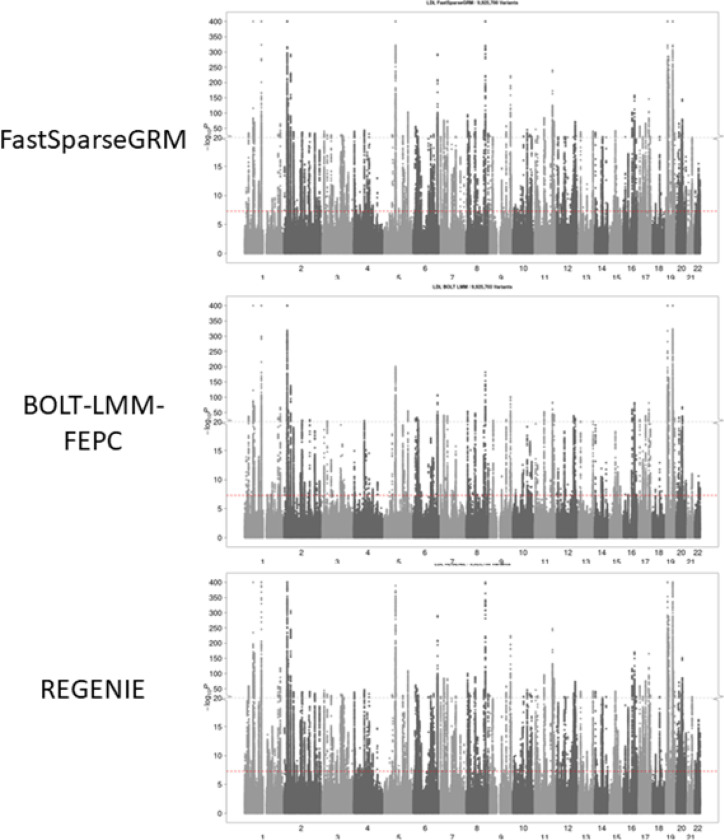
GWAS results for LDL (N = 452,476) from the entire UK Biobank data: Manhattan plots using FastSparseGRM, BOLT-LMM, and REGENIE. All methods were adjusted for age, age2, sex, age x sex, and 20 top ancestry PCs. ~ 9.9 million SNPs with imputation INFO >= 0.3 and minor allele frequency (MAF) >= 0.01 were analysed. BOLT-LMM-FEPC denotes BOLT-LMM method with ancestry PCs as fixed effects.

We applied our FastSparseGRM pipeline on the entire UK Biobank data and performed GWAS of five biomarkers: Trigycerides (N = 464,040), HDL (N = 425,076), LDL (N = 452,476), BMI (N = 485,332), and Total Bilirubin (N = 462,454). All methods were adjusted for age, age^2^, sex, age x sex, and 20 top ancestry PCs, and all phenotypes were rank-inverse normalized. BOLT-LMM included PCs as fixed effects since without PCs, BOLT-LMM can potentially have inflated type-I errors (see Simulation Studies). BOLT-LMM and REGENIE both used 599,685 array-genotyped SNPs (see Quality control and variant-filtering) as model SNPs and for the ridge regression step, respectively. Figure 3 shows the GWAS results for LDL on ~ 9.9 million SNPs with imputation INFO > = 0.3 and minor allele frequency (MAF) > = 0.01 using BOLT-LMM, REGENIE, and FastSparseGRM methods. fastGWA did not complete fitting the LMM null model fitting step within 1.5TB 5-day limit. The Manhattan plots showed agreement among all the methods, whereas FastSparseGRM required massively lower computation time to run step 1 (~ 6 minutes per phenotype on 1 CPU to fit the null model) compared to REGENIE (~ 90 minutes per phenotype on 30 CPUs) and BOLT-LMM (~ 344 minutes per phenotype on 30 CPUs), and similar computation time to run step 2 (genome-wide association tests). GWAS results for the other four biomarkers (Supplementary Fig. 2 for MAF > = 0.01 and Supplementary Fig. 3 for MAC > = 15) also demonstrate agreement among these methods.

### Gene-centric Analysis of Lipid traits from UK Biobank 200K WGS data

**Figure 4:**
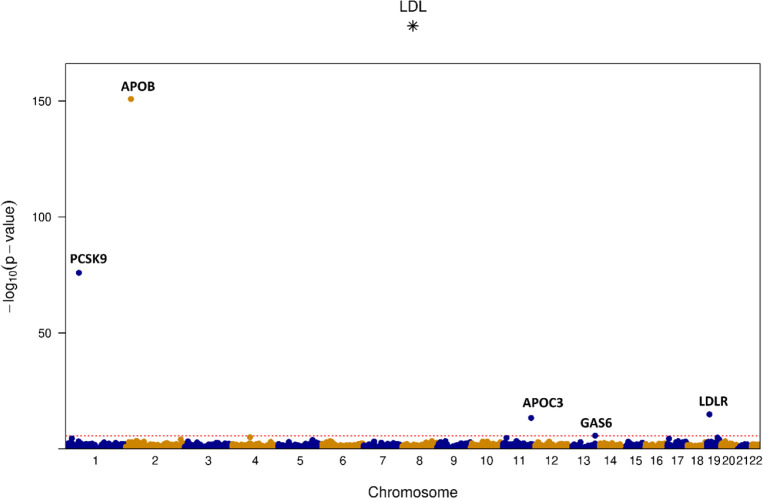
RVAS results for LDL (N = 185,346) from the UK Biobank 200K WGS data: Manhattan plots of gene-centric rare coding variant analysis of pLOF variants using the STAARpipeline with BDSA-GRM from FastSparseGRM. Analysis was adjusted for age, age2, sex, age x sex, and 20 top ancestry PCs.

Our BDSA-GRM readily fits into the STAARpipeline^13,27^, allowing it to be used also for rare variant WGS data analysis. STAAR can computationally benefit massively from using our BDSA-GRM. We demonstrate this by performing gene-centric rare (Minor Allele Frequency (MAF) < 0.01) coding rare variant analysis of the putative loss-of-function (pLOF) variants on the lipid traits (LDL: N = 185,346, HDL: N = 174,380, and Triglycerides: N = 189,951) on the 200K WGS data. Using our BDSA-GRM, the STAAR null model fitting only required 27–50 seconds on 1 CPU with 2.34 GB peak memory usage. In comparison, the null model fitting is not feasible with a dense GRM within a 5-day limit. Figure 4 shows the STAAR-O p-values for LDL (Supplementary Fig. 4 shows the results for the other two traits). Overall, STAARpipeline identified 17 significant genes across the three traits (Supplementary Table 2).

### Simulation Studies

**Figure 5:**
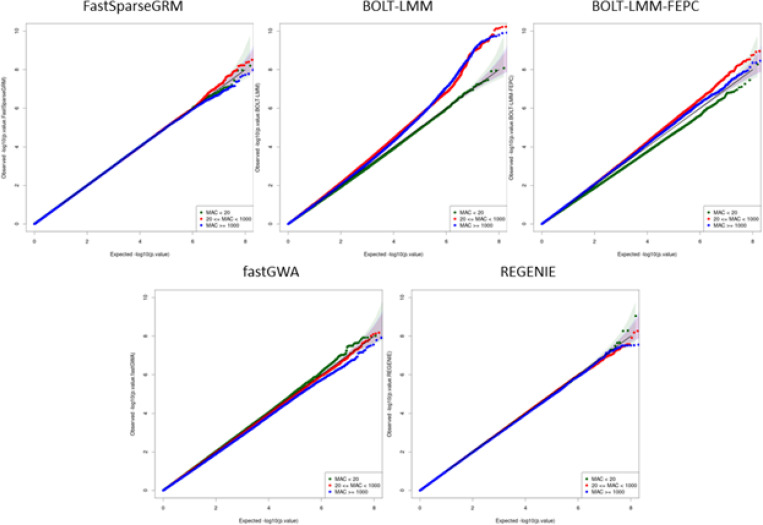
Quantile-quantile (QQ) plots of the p-values from FastSparseGRM, BOLT-LMM, BOLT-LMM-FEPC, fastGWA, and REGENIE: The p-values were obtained from genome-wide association analysis of 100 simulated traits. The traits were simulated based on ancestry effect $\text{AE}={{\text{h}}_{\text{ancestry}}}^{2}=0.2$ and polygenic effect $\text{PE}={{\text{h}}_{\text{polygenic}}}^{2}=0.4$. The QQ plots are also color-coded based on minor allele frequency (MAF) of the variants.

We first investigated the type I error rate calibration of FastSparseGRM, BOLT-LMM, fastGWA, and REGENIE using subset A (N=10,000) from the UK Biobank dataset. Subset A was chosen for this evaluation because it mimics a real-world multi-ancestry dataset with subjects having real-world population heterogeneity and sample-relatedness. Phenotypes were simulated using the real genotypes in the dataset using a polygenic effect model, and association tests were conducted on the imputed genetic markers in the UK Biobank (see Online Methods). BOLT-LMM was applied using both model specifications, with (BOLT-LMM-FEPC) and without PCs (BOLT-LMM) as fixed effects, where FEPC stands for fixed effects ancestry PCs. REGENIE, BOLT-LMM, BOLT-LMM-FEPC were applied using the leave-one-chromosome-out (LOCO) setting (default in both software). We observed that the p-values were well calibrated in FastSparseGRM and REGENIE, small amount of miscalibration was observed for BOLT-LMM-FEPC, whereas fastGWA and BOLT-LMM resulted in substantially conservative and anti-conservative p-values, respectively (Fig. 5 and Supplementary Fig. 5). The anti-conservativeness of BOLT-LMM demonstrates that not using PCs as fixed effects can potentially lead to false positives in association analysis on multi-ancestry datasets. We notice that since fastGWA does not adjust for population structure in its sparse GRM, the non-zero elements represent the population structure instead of the familial relatedness (Fig. 2). This leads to the highly conservative p-values in association tests when the ancestry effect is strong, even though fastGWA works well for mostly homogeneous populations. Moreover, since the non-zero elements in the fastGWA sparse GRM corresponds to larger ancestral population blocks instead of smaller family blocks, it also has much less sparsity than the BDSA-GRM (~ 2600 times less sparse), resulting in much higher computation time and memory required to fit the LMM (Supplementary Table 1).

Next, we evaluated the empirical power of FastSparseGRM, BOLT-LMM-FEPC, fastGWA, and REGENIE using subset A (N=10,000). We excluded the standard BOLT-LMM method which does not include PCs as fixed effects for this comparison as it showed substantially inflated type I errors, and only included BOLT-LMM-FEPC for the comparisons. Moreover, the empirical powers were calculated adjusting for the empirical type-I error levels so that the false positive rates (FPR) do not exceed 5 × 10^−8^ for any of the methods, i.e., if the FPR was larger than q 5 × 10^−8^ at type-I error level $\alpha =5\times 10^{-8}$, the type-I error level cutoff was increased to keep $FPR\leq 5\times 10^{-8}$. Variants were categorized based on how correlated their genotypes were to the population structure represented by the ancestry PCs (see Online Methods).

**Figure 6:**
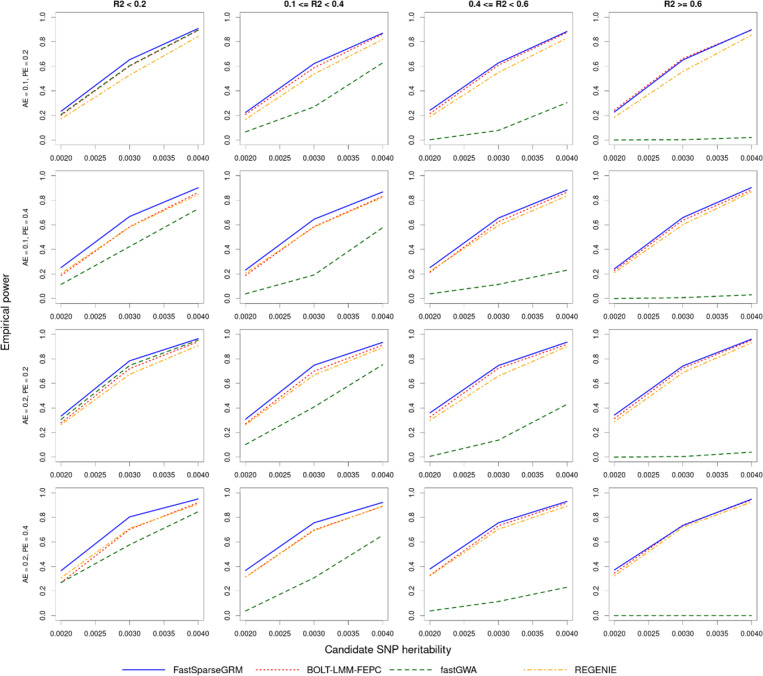
Empirical powers of FastSparseGRM, BOLT-LMM-FEPC, fastGWA, and REGENIE: Empirical powers were calculated based on 500 simulated trait-variant tests for each configuration of the ancestry effect $\left(\text{AE}={{\text{h}}_{\text{ancestry}}}^{2}\in \{0.1,0.2\}\right)$, polygenic effect $\left(\text{PE}={{\text{h}}_{\text{polygenic}}}^{2}\in \{0.2,0.4\}\right)$, and candidate SNP heritability $\left({{\text{h}}_{\text{SNP}}}^{2}\in \{0.002,0.003,0.004\}\right)$. The empirical type-I error level was adjusted to make sure the false positive rate does not exceed 5×10^(−8)^.

Across all different simulation settings and variant categories, FastSparseGRM retained the highest overall empirical powers (Fig. 6). REGENIE resulted in slightly lower empirical power compared to BOLT-LMM-FEPC across all scenarios. The power improvements of FastSparseGRM over BOLT-LMM-FEPC and REGENIE were more pronounced when the variants were nearly uncorrelated with the PCs $\left(R^{2}<0.2\right)$, and decreased as the variants became more strongly correlated with the ancestry PCs. For example, the improvements in power for FastSparseGRM over BOLT-LMM-FEPC and REGENIE were ~ 5–10% and ~ 9–13% respectively, when the variants had $R^{2}<0.2$ with the PCs and the SNP-heritability was $h_{SNP}^{2}=0.003$ (See Online Methods for details). For fastGWA, on the other hand, the power gap increased greatly as the variants became more correlated with the ancestry PCs, and fastGWA achieved in near zero empirical powers when the population structure had the strongest effects. These results suggest that by partitioning the population structure and relatedness, FastSparseGRM achieves accurate p-value calibration and improved empirical power to analyse multi-ancestry datasets.

## Discussion

We proposed a novel pipeline for scalable analysis of large multi-ancestry biobank data that accurately controls for population structure using ancestry PCs as fixed effects, and a BDSA-GRM to account for the sample relatedness. In addition to benefitting from parallel computation in high-performance computing clusters, our method FastSparseGRM provides substantially improved computational performance as it efficiently estimates the BDSA-GRM without requiring to compute a dense GRM first. Moreover, FastSparseGRM provides the ancestry PCs as a by-product of the pipeline, and thus removing the requirement to calculate them separately and enter them as input variables which most other existing methods require. FastSparseGRM is currently the only method that can calculate a BDSA-GRM for the entire UK Biobank dataset of 487,305 subjects using reasonable computing resources. The use of a BDSA-GRM in the FastSparseGRM pipeline further facilitates scalable estimation of the LMM parameters which involves repeated matrix-vector multiplications on the GRM.

We have thoroughly evaluated the performances of state-of-the-art methods (BOLT-LMM, REGENIE, fastGWA, and GENESIS) and compared them with FastSparseGRM using extensive simulation studies. Computationally, FastSparseGRM shows substantially improved performance in the GRM calculation and LMM fitting steps compared to all the existing methods. When p-value calibration and empirical powers are compared in multi-ancestry datasets, FastSparseGRM has unique benefits to each of the existing methods. FastSparseGRM and REGENIE resulted in well-calibrated p-values whereas the p-values showed substantial inflation or deflation in BOLT-LMM and fastGWA. Among the methods that showed fairly calibrated p-values, FastSparseGRM resulted in the greatest empirical powers across various simulation settings.

There are several limitations and scope for future research in our method. Firstly, in this paper, we used KING IBD segment method to identify related pairs because it is robust to both phased and unphased genotypes. However, if phased genotypes are available, then other IBD segment-based methods such as RAFFI^29^ and Truffle^30^ that leverages the phasing information, can potentially further reduce the computational time. In future, we plan to implement support for other IBD segment-based relationship inference methods and explicit knowledge of the pedigree, if available.

Secondly, we applied our method on the UK Biobank data to demonstrate the scalability. The UK Biobank, in particular, overwhelmingly consists of subjects with white British ancestry (408,582 subjects among 487,305 are white British). Therefore, to demonstrate the performance of our method in terms of p-value calibration and power, we used subsets of the UK Biobank data in the simulation studies to mimic the diversity of multi-ancestry studies.

Thirdly, even though, in this paper, we described the analysis pipeline for quantitative traits, the BDSA-GRM and the genetic PCs provided by FastSparseGRM can have wider applications in the analysis of non-quantitative traits. The GMMAT^12^ method, which allows the analysis of binary traits, have updated and implemented the use of our BDSA-GRM. In this paper, we have also demonstrated the use of our BDSA-GRM in the STAARpipeline for WGS rare variant association analysis, which can be readily applied to meta-analysis using MetaSTAAR^31^ and multi-trait analysis using MultiSTAAR^32^. Another popular analysis method, SAIGE^33^, allows uses to provide a sparse GRM. Our BDSA-GRM could potentially be plugged-in there as well.

Finally, in stratified cohorts, researchers might be interested in using multiple relatedness matrices instead of one single GRM to capture different nested or overlapping relationships among the subjects. Unlike BOLT-LMM and REGENIE, our LMM framework is robust to such requirements, and can handle multiple random effects and corresponding relatedness matrices. It is a potential future research interest to explore the benefits of such explicit modelling of complex relationship structures.

## Online Methods

### Calculation of the BDSA-GRM

**Figure 7:**
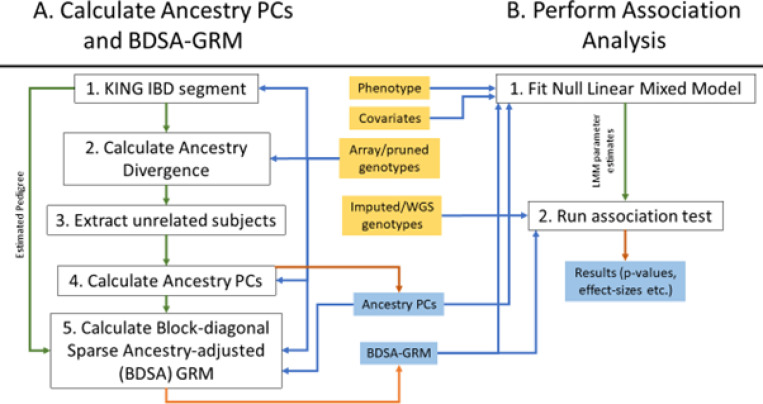
Schematic representation of the FastSparseGRM pipeline

The BDSA-GRM is calculated through five steps in FastSparseGRM as follows and its schematic representation is provided in Fig. 7,

#### Step 1 (IBD segment)

We start by applying KING IBD segment-based method to identify the sample pairs that are related up to a certain degree (default is 4). Even though the original KING method^26^ to calculate kinship is shown to be biased under population structure and admixture^18^, and sometimes can even result in negative kinship estimates, the recently developed IBD segment method implemented in KING can provide robust inference on the relatedness. As suggested in the KING software documentation, we do not use any allele frequency-based filtering or linkage disequilibrium (LD)-pruning of the genotyped SNPs for this step. We denote to be the set subjects that do not belong to any related pair, and to be the set of subjects that belong to at least one related pair.

#### Step 2 (Ancestry Divergence)

Next, we compute the ancestry divergence score^22^ for each subject in. Steps 2 and 3 follows the algorithm introduces in PC-AiR^22^. We will use them to select an ancestrally diverse subset of unrelated samples in step 3. Let be the genotype matrix for subjects and variants. The ancestry divergence score for subject is given by,

$${\text{A}}_{i}=\sum_{\underset{i^{\prime }\neq i}{i=1}}^{N}\delta_{\left\{K_{ii^{\prime }}<-\tau_{K}\right\}},\;K_{ii^{\prime }}=\frac{1}{2}\left(1-\frac{\sum_{j\in J_{i,i^{\prime }}}{\left(Z_{ij}-Z_{i^{\prime }j}\right)}^{2}}{\sum_{j\in J_{i,i^{\prime }}}\left(\delta \left\{z_{ij}=1\right\}+\delta \left\{z_{i^{\prime }j}=1\right\}\right)}\right),$$

where $\delta_{\left\{E\right\}}$ denotes the indicator function $\delta_{\left\{E\right\}}=1$ if E is true, or 0 otherwise, and $J_{i,i^{\prime }}$ denotes the set of variants with non-missing genotypes in both subjects i and $i^{\prime }$. To reduce the computation cost, we can use J=10,000 randomly selected variants that have non-missing genotypes in subjects i and $i^{\prime }$ to calculate the quantity $K_{ii^{\prime }}$ for large datasets. The value of $K_{ii^{\prime }}$ is close to 0 for a pair of unrelated subjects from similar ancestry, whereas the farther in ancestry the subjects i, $i^{\prime }$ are, the smaller the value of $K_{ii^{\prime }}$ becomes. Here $\tau_{K}$ is a suitable threshold such that $-\tau_{K}$ represents the expected lower bound of $K_{ii^{\prime }}$ for an unrelated pair of subjects i, $i^{\prime }$ from the same ancestry. In our implementation, we use $\tau_{K}=2^{-5.5}=0.022$ which is similar to the threshold used in PC-AiR $\left(\tau_{K}=0.025\right)$. The ancestry divergence score ${\text{A}}_{i}$ here represents the number of ancestrally dissimilar unrelated subjects to subject i. This quantity is used in step 3 to select more ancestrally diverse subjects in the set of unrelated subjects so that the population structure is more well-captured by PCA in step 4.

#### Step 3 (Extract Unrelated)

We use a greedy algorithm to partition the subjects into sets of unrelated and related subjects as follows,

Step 3a. Initialize $\text{U}={\text{R}}^{\left(0\right)}$ and R=φ, the null set. Through this algorithm, we will be moving subjects from U to R and stop when the remaining subjects in U are all unrelated among themselves.

Step 3b. Let ${\text{N}}_{i}$ be the number of subjects in U that also belong in a related pair with subject i, for each i∈U. If $\underset{j}{\max}{\text{N}}_{j}>0$, go to step 3c, otherwise, go to step 3g.

Step 3c. Get ${\text{M}}_{R}=\left\{i|{\text{N}}_{i}=\underset{j}{\text{max}}N_{j}\right\}$, the set of subjects with the greatest number of relatives in U. If $\left|{\text{M}}_{R}\right|=1$, we set $\text{M}={\text{M}}_{R}$ and move to step 3f, otherwise, go to step 3d.

Step 3d. Get ${\text{M}}_{A}=\left\{i|{\text{A}}_{i}=\underset{j\in {\text{M}}_{R}}{\min}{\text{A}}_{j}\right\}$, the set of subjects with the least ancestry divergence score. If $\left|{\text{M}}_{A}\right|=1$, we set $\text{M}={\text{M}}_{A}$ and move to step 3f, otherwise, go to step 3e.

Step 3e. Set M={i} where i is a randomly selected from ${\text{M}}_{A}$.

Step 3f. Update the sets U=U∖M, and R=R∪M, and return to step 3b.

Step 3g. Update $\text{U}=\text{U}\cup {\text{U}}^{\left(0\right)}$ and stop.

This algorithm is similar to the algorithm proposed in the PC-AiR method, except our algorithm does not include the KING kinship estimates which can be biased due to population structure and presence of admixed individuals. One can easily see that this algorithm results in a larger sample size and more ancestrally diverse subjects in set U compared to just selecting one subject from each related block of subjects and appending them to ${\text{U}}^{\left(0\right)}$.

#### Step 4 (Perform PCA)

We apply PCA on the genotypes of the subjects in to obtain the PC spaces (loadings), and then project the genotypes all the subjects on that PC space to calculate the PC scores. We developed a rapid parallelizable implementation of the fastPCA^25^ algorithm to perform this PCA step. Following the common practice for calculating ancestry PCs^15,18,34^ and GRMs^18^, we can use only LD-pruned common variants (MAF > 5%) for this step and step 5. Randomized PCA algorithms have been shown to be highly accurate and efficient when we are only interested in top few PCs^23–25^.

Let $Z=\left[\begin{array}{c} Z_{\text{U}}\\ Z_{\text{R}} \end{array}\right]$ be a partition of the genotype matrix, where $Z_{\text{U}}$ and $Z_{\text{R}}$ denote the $N_{\text{U}}\times M$ and $N_{\text{R}}\times M$ genotype matrices of the subjects belonging to the sets U and R, respectively. Here, $N_{\text{U}}$ and $N_{\text{R}}$ denote the cardinality of the sets U and R, respectively, and M is the number of variants used for the PCA. Let ${\tilde{Z}}_{\text{U}}$ and ${\tilde{Z}}_{\text{R}}$ denote the scaled-and-centered genotype matrices, i.e., ${\tilde{Z}}_{\text{U},ij}=\frac{Z_{\text{U},ij}-2{\hat{p}}_{j}}{\sqrt{2{\hat{p}}_{j}\left(1-{\hat{p}}_{j}\right)}}$ and ${\tilde{Z}}_{\text{R},ij}=\frac{Z_{\text{R},ij}-2{\hat{p}}_{j}}{\sqrt{2{\hat{p}}_{j}\left(1-{\hat{p}}_{j}\right)}}$, where ${\hat{p}}_{j}=\frac{\underset{i}{\sum }Z_{\text{U},ij}}{2N_{\text{U},j}}$ is the estimated minor allele frequency (MAF) based on the unrelated Subjects, and $N_{\text{U},j}$ is the number of non-missing genotypes for the *j*-th variant among the subjects in U. Further, partition ${\tilde{Z}}_{\text{U}}=\left[\begin{array}{c} {{\tilde{Z}}_{\text{U}}}^{\left(1\right)}\;\ldots \;{{\tilde{Z}}_{\text{U}}}^{\left(C\right)} \end{array}\right]$ into C blocks, i.e., split SNPs into C blocks with each ${{\tilde{Z}}_{\text{U}}}^{\left(c\right)}$ having the dimension N×M/C, so that those can be processed parallelly in C computing cores. Suppose we are interested in obtaining the top K PCs. Then, we start with a random N×L matrix $H_{0}$ entries generated from N(0,1). As suggested by Galinsky et al.^25^, We choose L to be equal to twice the number of PCs We are interested in. Then iteratively, in the r=1,…,R-th iteration, we compute,

$${\tilde{H}}_{r}={{\tilde{Z}}_{\text{U}}}^{⊤}H_{r}=\left[\begin{array}{c} {{\tilde{Z}}_{\text{U}}}^{(1)⊤}\;H_{r}\\ \vdots \\ {{\tilde{Z}}_{\text{U}}}^{(C)⊤}\;H_{r} \end{array}\right],\;\text{and}\;H_{r+1}={\tilde{Z}}_{\text{U}}{\tilde{H}}_{r}=\sum_{c=1}^{C}{{\tilde{Z}}_{\text{U}}}^{\left(c\right)}{{\tilde{H}}_{r}}^{\left(c\right)},$$

where ${\tilde{H}}_{r}^{\left(c\right)}={{\tilde{Z}}_{\text{U}}}^{(c)⊤}H_{r}$. Each computation of ${{\tilde{Z}}_{\text{U}}}^{(c)⊤}H_{r}$ and ${{\tilde{Z}}_{\text{U}}}^{\left(c\right)}{{\tilde{H}}_{r}}^{\left(c\right)}$ can be performed parallelly. Galinsky et al. also showed that R=10 iterations are enough to provide accurate PCA using this method.

After the iterative steps, we aggregate the ${\tilde{H}}_{r}$ matrices into the M×(R+1)L matrix $\tilde{H}=\left[\begin{array}{c} {\tilde{H}}_{0}\;\ldots \;{\tilde{H}}_{R} \end{array}\right]$, and apply singular value decomposition (SVD) on $\tilde{H}$. Let the SVD be $\tilde{H}=P_{H}D_{H}Q_{H}^{⊤}$. Next, we construct the N×(R+1)L matrix $U={\tilde{Z}}_{\text{U}}P_{H}$ and perform another SVD on U, which we denote by $U=P_{U}D_{U}Q_{U}^{⊤}$. Then the first K column vectors of $P_{U}$ will provide the estimates for the top K PC scores of $\tilde{Z}$. Notice that, the matrices $\tilde{H}$ and U have dimensions M×(R+1)L and N×(R+1)L, respectively, which are substantially smaller than the dimension of the genotype matrix ${\tilde{Z}}_{\text{U}}$, and therefore the SVD computations can be performed efficiently. The PC scores for the subjects in R can then be calculated by $P_{\text{R}}^{\left(K\right)}=\frac{{\tilde{Z}}_{\text{R}}{{\tilde{Z}}_{\text{U}}}^{⊤}P_{\text{U}}^{\left(K\right)}}{\left\|{{\tilde{Z}}_{\text{U}}}^{⊤}P_{\text{U}}^{\left(K\right)}\right\|}$, where $P_{\text{U}}^{\left(K\right)}$ is the N×K matrix comprised of only the first K columns of $P_{U}$. Therefore, the top K ancestry PCs for all the subjects are given by $P^{\left(K\right)}=[\begin{array}{c} P_{\text{U}}^{\left(K\right)}\\ P_{\text{R}}^{\left(K\right)} \end{array}]$ where $P_{\text{U}}^{\left(K\right)}$ and $P_{\text{R}}^{\left(K\right)}$ corresponds to subjects in the sets U and R.

Our parallelized PCA algorithm requires only $O\left(\frac{NM}{C}\right)$ user time, where N, M, and C are sample-size, number of variants, and number of computing cores, respectively. Moreover, we implemented reading and processing the genotype data in 2-bit format, and use bitwise operations to achieve computational efficiency.

#### Step 5 (Calculate BDSA-GRM)

The final step in this algorithm involves obtaining the ancestry-adjusted allele frequencies for each subject and using them to calculate the BDSA-GRM. We first obtain the expected ancestry-adjusted allele frequencies as for each subject, which we estimate by training a linear regression model of the genotypes on the PCs for each marker. To ensure the estimates lies within 0 and 1, we replace the estimates with or when or, respectively for a very small value. Using the fitted linear regression coefficients, we predict the expected ancestry-adjusted allele frequencies for all the subjects.

Next, we construct the sparse GRM by only estimating the non-zero elements. Specifically, we create a network graph G=(V,E) of the subjects where each subject i is a vertex (i∈V), and each edge (i,j)∈E represents the subjects i and j to be related as determined by KING IBD segment method from step 1. Based on the constructed graph G, we cluster the subjects into disjoint connected components, i.e., partition G into connected components ${\text{G}}_{1}=\left({\text{V}}_{1},{\text{E}}_{1}\right),\ldots ,{\text{G}}_{P}=\left({\text{V}}_{P},{\text{E}}_{P}\right)$ such that ${\text{V}}_{p}\cap {\text{V}}_{p^{\prime }}=\varphi$ for any $1\leq p\neq p^{\prime }\leq P$. Then, for each subgraph ${\text{G}}_{P}$, we estimate the elements of the sparse GRM Ψ as,

$${\text{Ψ}}_{i,i^{\prime }}=\frac{\sum_{j\in J_{i,i^{\prime }}}\left(Z_{ij}-2{\hat{p}}_{ij}\right)\left(Z_{i^{\prime }j}-2{\hat{p}}_{i^{\prime }j}\right)}{4{\sum_{j\in J_{i,i^{\prime }}}\left[{\hat{p}}_{ij}\left(1-{\hat{p}}_{ij}\right){\hat{p}}_{i^{\prime }j}\left(1-{\hat{p}}_{i^{\prime }j}\right)\right]}^{\frac{1}{2}}},$$

for all $i,i^{\prime }\in {\text{V}}_{p}$. Here $J_{i,i^{\prime }}$ denotes the set of variants with non-missing genotypes in both subjects i and $i^{\prime }$. To efficiently compute these estimates using parallel computing on multiple cores, in our software, we split the genome-wide SNPs into smaller chunks and process them parallelly to calculate the numerator and the denominator terms. Depending on the available memory, the chunk sizes can also be customized. Once we have obtained ${\text{Ψ}}_{i,i^{\prime }}$ for all pairs or subjects in all of the components, we set the rest of the elements of the Ψ matrix to be zero. Finally, we rearrange the subjects (therefore rows and columns of Ψ) to make Ψ block-diagonal by keeping the subjects within each component ${\text{G}}_{p}$ contiguous.

### Single-variant association analysis model

To test for genotype-phenotype associations, we apply the following LMM,

Y=Xβ+Gγ+b+є,

for N subjects, where $Y={\left(Y_{1},\ldots ,Y_{N}\right)}^{⊤}$ is the N×1 trait outcome vector, $X=\left(X_{1}^{⊤},\ldots X_{N}^{⊤}\right)$ is the N×k covariate matrix that includes the intercept and the genetic PCs, $G={\left(G_{1},\ldots ,G_{N}\right)}^{⊤}$ is the N×1 genotype vector for the variant being tested, β and γ are corresponding coefficient parameters, b is a random effect vector to model the familial relatedness among the subjects, and є is the random environmental effect. We assume that $\text{є}={\left({\text{є}}_{1},\ldots ,{\text{є}}_{N}\right)}^{⊤}∼N\left(0,\sigma^{2}I\right)$, and $b={\left(b_{1},\ldots ,b_{N}\right)}^{⊤}∼N\left(0,\tau^{2}\Phi \right)$, where I denotes the N×N identity matrix, Φ denotes the BDSA-GRM, and $\sigma^{2}$ and $\tau^{2}$ are corresponding variance component parameters. This results in the marginal variance of Y to be $\Sigma =\sigma^{2}I+\tau^{2}\Phi$.

We perform the analysis in two steps: 1) fit the null model without the target variant, 2) perform a score test for the association between the genotypes of the target variant and the phenotype using the parameter estimates from the null model.

#### Step 1 (fitting the null model)

Given the variance component parameters, the best linear unbiased estimates and predictors (BLUE and BLUP) for and respectively can be obtained from the null model,

Y=Xβ+b+є,

using Henderson’s mixed model equations^12,35^,

$$\left[\begin{array}{cc} X^{⊤}X & X^{⊤}\\ X & I+\frac{\sigma^{2}}{\tau^{2}}\Phi^{-1} \end{array}\right]\left[\begin{array}{c} \hat{\beta }\\ \hat{b} \end{array}\right]=\left[\begin{array}{c} X^{⊤}Y\\ Y \end{array}\right].$$

The solutions to these equations provide the estimates/predictors,

$$\hat{\beta }={\left(X^{⊤}{\text{Σ}}^{-1}X\right)}^{-1}X^{⊤}{\text{Σ}}^{-1}Y,\;\hat{b}=\left(\tau^{2}\Psi \right){\text{Σ}}^{-1}(Y-X\hat{\beta }).$$

To estimate the variance component parameters, we use the following restricted likelihood^12^,

$$I_{R}\left(\sigma^{2},\tau^{2}\right)=C-\frac{1}{2}\log|\Sigma |-\frac{1}{2}\log|X^{⊤}\Sigma^{-1}X|-\frac{1}{2}Y^{⊤}PY,$$

where C is invariable with respect to $\left(\sigma^{2},\tau^{2}\right)$, and $P={\text{Σ}}^{-1}-{\text{Σ}}^{-1}X{(X^{⊤}{\text{Σ}}^{-1}X)}^{-1}X^{⊤}{\text{Σ}}^{-1}$. We maximize this likelihood using the Average Information Restricted Maximum Likelihood (AI-REML)^12,36^ algorithm, where the score vector and the average information are given by,

$$\nabla =\left[\begin{array}{c} \frac{1}{2}\left\{Y^{⊤}P^{2}Y-\mathrm{tr}(P)\right\}\\ \frac{1}{2}\left\{Y^{⊤}P\text{Φ}PY-\mathrm{tr}(P\text{Φ})\right\} \end{array}\right],\;AI=\left[\begin{array}{cc} \frac{1}{2}Y^{⊤}P^{3}Y & \frac{1}{2}Y^{⊤}P^{2}\Phi PY\\ \frac{1}{2}Y^{⊤}P\Phi P^{2}Y & \frac{1}{2}Y^{⊤}{\left(P\Phi \right)}^{2}PY \end{array}\right].$$

#### Step 2 (score test)

Once we have obtained the null model parameters, we can perform a genome-wide scan of variants using the score test. For a variant with genotype vector, the score test statistic and the variance of the test statistic under the null hypothesis of no association will be,

$$T=G^{⊤}PY,\;Var_{H_{0}}(T)=G^{⊤}PG.$$

Computing this variance for every single variant can be computationally burdensome as it involves multiplications of the large dimensional P matrix. To achieve fast computation of the score test genome-wide, we use the variance-ratio approximation commonly used in BOLT-LMM and fastGWA, where the variance of the score statistic is first calculated assuming the subjects are independent, and then adjusted by a variance-ratio^28^ factor that is calculated based on a small number (100) of variants.

### Data Simulation

We conducted extensive simulation studies to assess the p-value calibration and power of the FastSparseGRM pipeline and compare them to BOLT-LMM, REGENIE, and fastGWA. For subjects i=1,…,N, we simulated the phenotypes following,

$$Y_{i}=X_{i}^{⊤}\beta_{ancestry}+\sum J_{j=1}^{J}Z_{ij}\beta_{polygenic,j}+G_{i}\beta_{SNP}+{\text{є}}_{i},$$

where $X_{i}$ is a q×1 vector (q=10,20) representing the top 10 (for subsets A, and B) or 20 (for subset C, and the entire UK Biobank data) ancestry PCs, $Z_{ij}$ are real observed genotypes for J=20,000 randomly selected SNPs (MAF>5%, only on odd autosomes), $G_{i}$ is the genotype of the SNP that is being tested (only on even autosomes to make sure it is not in LD with any of the SNPs in Z), and ${\text{є}}_{i}∼N\left(0,\sigma^{2}\right)$ iid are residual environmental effects. The parameters $\beta_{ancestry},\beta_{polygenic,j},\beta_{SNP}$, and $\sigma^{2}$ were selected to ensure that the proportion of variance in the phenotype explained by each of these four components: ancestry, polygenic effect, SNP effect, and environmental effect were equal to $h_{ancestry}^{2},h_{polygenic}^{2}h_{SNP}^{2}$, and $h_{environmental}^{2}$. We note that, the effect of the sample-relatedness is attributed to the polygenic effect here, as the SNPs are related among related subjects, and since we are using the real genotypes from the UK Biobank subjects, our analysis incorporates real-world genetic relatedness among subjects. We can interpret these four components as the traditionally regarded heritability components.

For both p-value calibration and power analysis, we considered $h_{ancestry}^{2}\in \{0.1,0.2\}$, and $h_{polygenic}^{2}\in \{0.2,0.4\}$. The SNP effect $h_{SNP}^{2}$ was set at 0 for the calibration analysis, and $h_{SNP}^{2}\in \{0.002,0.003,0.004\}$ for power analysis. The environmental component


$$h_{environmental}^{2}=1-(h_{ancestry}^{2}+h_{polygenic}^{2}+h_{SNP}^{2}).$$

To assess the p-value calibration, we simulated 100 phenotypes and tested ~ 10.98 million imputed SNPs (MAC>15, INFO≥0.3, on the even-numbered autosomes to make sure they are not in LD with the SNPs that had polygenic effects on the phenotype) against each of them, resulting in ~ 10^9^ total tests for each specification of $h_{ancestry}^{2}$ and $h_{polygenic}^{2}$.

To assess the empirical power, we first categorized the SNPs (on the even-numbered autosomes) based on how correlated they were with the ancestry PCs. For each SNP, we first calculated the multiple linear regression $R^{2}$ from the regression $G_{i}∼X_{i}^{⊤}\beta +\tau_{i}$, and then partitioned them into four categories, A) $R^{2}<0.2$, B) $0.2\leq R^{2}<0.4$, C) $0.4\leq R^{2}<0.6$, and D) $R^{2}\geq 0.6$. Next, from each category, we selected 50 SNPs randomly, and simulated 10 phenotypes for each of them, resulting in 500 total tests to assess the empirical power in each category and each specification of $h_{ancestry}^{2}$, $h_{polygenic}^{2}$, and $h_{SNP}^{2}$.

### Quality control and variant-filtering

We first performed filtering on the genotyped variants. For each of the subsets A, B, C, and the entire UK Biobank dataset, we first removed monomorphic variants, and all genotyped variants which failed any of the quality control tests conducted by the UK Biobank researchers (https://biobank.ctsu.ox.ac.uk/crystal/refer.cgi?id=1955) or had more than 10% missingness. We will denote these variants are Stage 1 variants. For subsets A, B, C, and the entire UK Biobank dataset, the number of Stage 1 variants were 590605, 594574, 594760, and 599685, respectively. Next, we removed the variants with MAF≤0.05 and LD-pruned the rest based on pairwise $R^{2}>0.2$. Due to the smaller sample-size in subset A, the MAF-based filtering criteria was relaxed and we only removed variants with MAF≤0.01 to make sure we retain enough variants for the downstream analysis. We denote the filtered variants as Stage 2 variants. For subsets A, B, C, and the entire UK Biobank dataset, the number of Stage 2 variants were 172045, 121114, 138608, and 124202, respectively. The Stage 1 variants were used to construct the sparse GRM using fastGWA, and to fit the null models (step 1) in BOLT-LMM and REGENIE. The Stage 2 variants were used to construct the BDSA-GRMs using FastSparseGRM and GENESIS. We performed sensitivity analysis to show that that the resulting BDSA-GRMs remain almost identical between using Stage 1 and Stage 2 variants.

We only considered imputed SNPs with INFO≥0.3 and minor allele count, MAC>15 for performing GWAS in both simulation studies and real-data examples. For the simulation studies, 10,980,662 autosomal SNPs on the even-numbered chromosomes were used for the tests, whereas for the real-data examples, ~ 9.9 million genome-wide SNPs (INFO > = 0.3, MAF > = 0.01) on all autosomes were used.

### Phenotype pre-processing

The phenotypes Triglycerides, HDL, LDL, BMI, and Total Bilirubin were curated from the UK Biobank fields 30870, 30760, 30780, 21001, and 30840 respectively. LDL values were either directly measured or calculated from adjusted total cholesterol levels using the Friedewald equation. LDL values were removed from analyses when triglycerides were > 400 mg/dl or when LDL was < 10 mg/dl. Considering the average effect of statins, when statins were present in the treatment list, LDL was adjusted by dividing by 0.7^13^. All phenotypes were then rank-inverse normalized before applying the GWAS and RVAS.

### Sensitivity analyses

Using subsets A, B, C, and the entire UK Biobank dataset (N=487,305), we performed sensitivity analyses to assess the robustness of the BDSA-GRM estimation with different specifications of variants used for Step 1 (IBD segment) and Step 2 (Ancestry Divergence). For Step 1, we compared between Stage 1 (no LD-pruning or MAF-filtering) and Stage 2 (LD-pruned and MAF-filtered) variants being used. For Step 2, we compared among using all the available filtered SNPs, and two sets of randomly selected 10,000 SNPs with different seeds. The comparison of the estimated BDSA-GRMs among these six configurations (two for Step 1 and three for Step 2) suggest that the method is robust to these specifications. The estimated non-zero values were almost identical among the six configurations across all of the subsets. To compare the coordinates of the elements that were thresholded to zero among different BDSA-GRMs, we calculated pairwise Jaccard’s distances and dissimilarity proportions. Across all of the subsets, all pairwise Jaccard’s distances and dissimilarity proportions were almost identical to zero, suggesting that the thresholded coordinates were almost identical across the six configurations (See Supplementary Fig. 6).

### Genome build

The genomic coordinates reported in this paper were based on NCBI Build 37/UCSC hg19.

## Supplementary Material

Supplement 1

## Figures and Tables

**Table 1:** Schematic representation of resource usage at different steps by popular LMM/RR-based GWAS methods: Computational complexity (number of additions and multiplications) and memory usage per SNP-phenotype association tested are listed in terms of *N* = number of subjects, *M* = number of SNPs used to calculate GRM or to fit the Null LMM, *S*^2^ = number of non-zero elements in the sparse GRM, *L* = size of the largest related subject-block in the sparse GRM, and *B* = number of SNPs in each block for the Ridge 0 step. Standard LMM represents the LMM method which is fitted using an unadjusted empirical GRM, such as implemented in the GCTA or GMMAT software. The number 3 in the expression of the computation time for the GENESIS method (with both dense and sparse GRMs) is used to emphasize the fact that the GRM calculation involves three computationally challenging sub-steps, each of which has a complexity of $O\left(N^{2}M\right)$, as opposed to standard LMM and fastGWA where only one such sub-step is required.

Resource	Step	Standard LMM (with Unadjusted Dense GRM)	GENESIS (with PC-Relate Dense GRM)	BOLT-LMM	fastGWA	GENESIS (with PC-Relate Sparse GRM)	REGENIE	FastSparseGRM
Computation Time	GRM calculation	$N^{2}M$	$3N^{2}M$		$N^{2}M$	$3N^{2}M$		$L^{2}M+NM$
	Null LMM/Ridge 0 + 1	$N^{3}$	$N^{3}$	$N^{1.5}M$	$S^{2}+N$	$L^{2}+N$	$NMB+MB^{2}+N{\left(\begin{array}{c} M\\ \bar{B} \end{array}\right)}^{2}+{\left(\begin{array}{c} M\\ \bar{B} \end{array}\right)}^{3}$	$L^{2}+N$
	Genome-wide Scan	$N^{2}$	$N^{2}$	N	N	$L^{2}+N$	N	N
Memory	GRM calculation	$N^{2}$	$N^{2}$		$N^{2}$	$N^{2}$		$NM+L^{2}$
	Null LMM/Ridge 0 + 1	$N^{2}$	$N^{2}$	NM	$S^{2}+N$	$L^{2}+N$	NM	$L^{2}+N$
	Genome-wide Scan	$N^{2}$	$N^{2}$	N	N	$L^{2}+N$	N	N

**Table 2:** Computation time and memory requirements for calculating the BDSA-GRM using FastSparseGRM and GENESIS methods: All computations were performed on 30 CPUs. To calculate the BDSA-GRMs, ~ 120,000–170,000 SNPs were used (detailed quality control procedures are in the Online Methods section). For the entire UK Biobank dataset, GENESIS did not complete (DNC) the calculation within the 5-day, 1.5TB constraint.

UK Biobank Subset	FastSparseGRM		GENESIS	
	Time (Hours)	Memory (GB)	Time (Hours)	Memory (GB)
A (N = 10,000)	0.05	7.1	0.66	60.8
B (N = 50,000)	0.13	32.7	19.90	390
C (N = 50,000)	0.16	36.4	18.82	390
Entire UK Biobank (N = 487,305)	10.73	113	DNC	DNC

## Data Availability

Individual-level genotype and phenotype data from the UK Biobank are available from http://www.ukbiobank.ac.uk. A formal application to the UK Biobank is required to download the data.

## References

[1] Bycroft C. The UK Biobank resource with deep phenotyping and genomic data. Nature 562, 203–209 (2018). 30305743 10.1038/s41586-018-0579-z PMC6786975

[2] Taliun D. Sequencing of 53,831 diverse genomes from the NHLBI TOPMed Program. Nature 590, 290–299 (2021). 33568819 10.1038/s41586-021-03205-y PMC7875770

[3] Mardis E.R. Next-generation DNA sequencing methods. Annu Rev Genomics Hum Genet 9, 387–402 (2008). 18576944 10.1146/annurev.genom.9.081307.164359

[4] Sirugo G., Williams S.M. & Tishkoff S.A. The Missing Diversity in Human Genetic Studies. Cell 177, 26–31 (2019). 30901543 10.1016/j.cell.2019.02.048 PMC7380073

[5] Gaziano J.M. Million Veteran Program: A mega-biobank to study genetic influences on health and disease. Journal of Clinical Epidemiology 70, 214–223 (2016). 26441289 10.1016/j.jclinepi.2015.09.016

[6] Bick A.G. Genomic data in the All of Us Research Program. Nature 627, 340–346 (2024). 38374255 10.1038/s41586-023-06957-x PMC10937371

[7] Investigators T.A.o.U.R.P. The “All of Us” Research Program. New England Journal of Medicine 381, 668–676 (2019). 31412182 10.1056/NEJMsr1809937 PMC8291101

[8] Loh P.-R. Efficient Bayesian mixed-model analysis increases association power in large cohorts. Nature Genetics 47, 284–290 (2015). 25642633 10.1038/ng.3190 PMC4342297

[9] Jiang L. A resource-efficient tool for mixed model association analysis of large-scale data. Nature Genetics 51, 1749–1755 (2019). 31768069 10.1038/s41588-019-0530-8

[10] Yang J., Zaitlen N.A., Goddard M.E., Visscher P.M. & Price A.L. Advantages and pitfalls in the application of mixed-model association methods. Nature genetics 46, 100–106 (2014). 24473328 10.1038/ng.2876 PMC3989144

[11] Kang H.M. Variance component model to account for sample structure in genome-wide association studies. Nature Genetics 42, 348–354 (2010). 20208533 10.1038/ng.548 PMC3092069

[12] Chen H. Control for Population Structure and Relatedness for Binary Traits in Genetic Association Studies via Logistic Mixed Models. Am J Hum Genet 98, 653–66 (2016). 27018471 10.1016/j.ajhg.2016.02.012 PMC4833218

[13] Li X. Dynamic incorporation of multiple in silico functional annotations empowers rare variant association analysis of large whole-genome sequencing studies at scale. Nature Genetics 52, 969–983 (2020). 32839606 10.1038/s41588-020-0676-4 PMC7483769

[14] Selvaraj M.S. Whole genome sequence analysis of blood lipid levels in > 66,000 individuals. Nature Communications 13, 5995 (2022). 10.1038/s41467-022-33510-7 PMC9553944 36220816

[15] Price A.L. Principal components analysis corrects for stratification in genome-wide association studies. Nature Genetics 38, 904–909 (2006). 16862161 10.1038/ng1847

[16] Yang J., Lee S.H., Goddard M.E. & Visscher P.M. GCTA: a tool for genome-wide complex trait analysis. Am J Hum Genet 88, 76–82 (2011). 21167468 10.1016/j.ajhg.2010.11.011 PMC3014363

[17] Gogarten S.M. Genetic association testing using the GENESIS R/Bioconductor package. Bioinformatics 35, 5346–5348 (2019). 31329242 10.1093/bioinformatics/btz567 PMC7904076

[18] Conomos M.P., Reiner A.P., Weir B.S. & Thornton T.A. Model-free Estimation of Recent Genetic Relatedness. Am J Hum Genet 98, 127–48 (2016). 26748516 10.1016/j.ajhg.2015.11.022 PMC4716688

[19] Mbatchou J. Computationally efficient whole-genome regression for quantitative and binary traits. Nature Genetics 53, 1097–1103 (2021). 34017140 10.1038/s41588-021-00870-7

[20] Tsuruta S., Misztal I. & Stranden I. Use of the preconditioned conjugate gradient algorithm as a generic solver for mixed-model equations in animal breeding applications. Journal of animal science 79, 1166–1172 (2001). 11374535 10.2527/2001.7951166x

[21] Conomos M.P., Reiner A.P., McPeek M.S. & Thornton T.A. Genome-Wide Control of Population Structure and Relatedness in Genetic Association Studies via Linear Mixed Models with Orthogonally Partitioned Structure. bioRxiv, 409953 (2018).

[22] Conomos M.P., Miller M.B. & Thornton T.A. Robust Inference of Population Structure for Ancestry Prediction and Correction of Stratification in the Presence of Relatedness. Genetic epidemiology 39, 276–293 (2015). 25810074 10.1002/gepi.21896 PMC4836868

[23] Halko N., Martinsson P.-G., Shkolnisky Y. & Tygert M. AN ALGORITHM FOR THE PRINCIPAL COMPONENT ANALYSIS OF LARGE DATA SETS. SIAM journal on scientific computing 33, 2580–2594 (2011).

[24] Halko N., Martinsson P.G. & Tropp J.A. Finding Structure with Randomness: Probabilistic Algorithms for Constructing Approximate Matrix Decompositions. SIAM review 53, 217–288 (2011).

[25] Galinsky K.J. Fast Principal-Component Analysis Reveals Convergent Evolution of ADH1B in Europe and East Asia. Am J Hum Genet 98, 456–472 (2016). 26924531 10.1016/j.ajhg.2015.12.022 PMC4827102

[26] Manichaikul A. Robust relationship inference in genome-wide association studies. BIOINFORMATICS 26, 2867–2873 (2010). 20926424 10.1093/bioinformatics/btq559 PMC3025716

[27] Li Z. A framework for detecting noncoding rare-variant associations of large-scale whole-genome sequencing studies. Nature Methods 19, 1599–1611 (2022). 36303018 10.1038/s41592-022-01640-x PMC10008172

[28] Svishcheva G.R., Axenovich T.I., Belonogova N.M., van Duijn C.M. & Aulchenko Y.S. Rapid variance components-based method for whole-genome association analysis. Nat Genet 44, 1166–70 (2012). 22983301 10.1038/ng.2410

[29] Naseri A., Shi J., Lin X., Zhang S. & Zhi D. RAFFI: Accurate and fast familial relationship inference in large scale biobank studies using RaPID. PLoS Genet 17, e1009315 (2021). 33476339 10.1371/journal.pgen.1009315 PMC7853505

[30] Dimitromanolakis A., Paterson A.D. & Sun L. Fast and Accurate Shared Segment Detection and Relatedness Estimation in Un-phased Genetic Data via TRUFFLE. Am J Hum Genet 105, 78–88 (2019). 31178127 10.1016/j.ajhg.2019.05.007 PMC6612710

[31] Li X. Powerful, scalable and resource-efficient meta-analysis of rare variant associations in large whole genome sequencing studies. Nature Genetics 55, 154–164 (2023). 36564505 10.1038/s41588-022-01225-6 PMC10084891

[32] Li X. A statistical framework for powerful multi-trait rare variant analysis in large-scale whole-genome sequencing studies. bioRxiv, 2023.10.30.564764 (2023). 10.1038/s43588-024-00764-8 PMC11981678 39920506

[33] Zhou W. SAIGE-GENE + improves the efficiency and accuracy of set-based rare variant association tests. Nature Genetics 54, 1466–1469 (2022). 36138231 10.1038/s41588-022-01178-w PMC9534766

[34] Patterson N., Price A.L. & Reich D. Population structure and eigenanalysis. PLoS genetics 2, e190–e190 (2006). 17194218 10.1371/journal.pgen.0020190 PMC1713260

[35] Verbeke G. Linear mixed models for longitudinal data, (Springer, New York, 2009).

[36] Gilmour A.R., Thompson R. & Cullis B.R. Average Information REML: An Efficient Algorithm for Variance Parameter Estimation in Linear Mixed Models. Biometrics 51, 1440–1450 (1995).

