# Supplementary material for: Scalable analysis of large multi-ancestry biobanks by leveraging sparse ancestry-adjusted sample-relatedness
Source: Res Sq. 2024 Nov 12:rs.3.rs-5343361. Preprint. [Version 1] doi: 10.21203/rs.3.rs-5343361/v1 (PMC11601839; doi:10.21203/rs.3.rs-5343361/v1)
Supplement: Supplement 1 [file NIHPPRS5343361V1-supplement-1.pdf]

Supplementary Files

This is a list of supplementary files associated with this preprint. Click to download.

- SupplementarysGRMtestSubmission.docx
